# Evaluation of fidaxomicin use in community hospitals after a clinical guideline change at a large health system and opportunities for stewardship

**DOI:** 10.1017/ice.2021.456

**Published:** 2023-02

**Authors:** Tina M. Khadem, M. Hong Nguyen, J. Ryan Bariola

**Affiliations:** Division of Infectious Diseases, Department of Medicine, University of Pittsburgh School of Medicine, Pittsburgh, Pennsylvania

**Keywords:** fidaxomicin, antimicrobial stewardship

## Abstract

After updating our health system’s *Clostridioides difficile* treatment protocols in 2018, we reviewed 104 unique hospital encounters involving fidaxomicin at 10 community hospitals. Half (50%) of regimens were adherent to our guidelines, with infectious diseases (ID) providers were frequently nonadherent. Antimicrobial stewardship programs are important for facilitating best practices, even among ID specialists.

Dissemination of clinical guideline changes within a health system is challenging. In April 2018, The University of Pittsburgh Medical Center (UPMC) updated institutional *Clostridioides difficile* infection (CDI) guidelines based partly on 2017 Infectious Diseases Society of America/Society for Healthcare Epidemiology of America (IDSA/SHEA) recommendations that suggested fidaxomicin or oral vancomycin to treat initial CDI.^1^ UPMC previously restricted fidaxomicin to second or subsequent recurrences and required infectious diseases (ID) or antimicrobial stewardship approval. After April 2018, UPMC allowed fidaxomicin or a tapering course of oral vancomycin for first or subsequent recurrences. Other fidaxomicin use was restricted to ID, gastroenterology (GI), or with approval by local hospital antimicrobial stewardship programs or local pharmacy and therapeutics committee (P&T) chairperson. We still restricted fidaxomicin in initial CDI to ensure continuity of postdischarge treatment because outpatient fidaxomicin cost remained a barrier.

Previous studies have evaluated the impact of IDSA/SHEA guidelines on CDI treatment.^2,3^ One outpatient prescription review found oral vancomycin and fidaxomicin use significantly increased while metronidazole use decreased following publication of the revised guidelines.^2^ In another study of community-onset CDI, metronidazole was still used in nearly 25% of cases.^3^ In an earlier UPMC review, oral vancomycin and fidaxomicin use increased and oral metronidazole slightly decreased after our institutional guidelines changed,^4^ but fidaxomicin use remained low compared to oral vancomycin. To better understand fidaxomicin prescribing at UPMC after our guideline change, we evaluated fidaxomicin orders from 10 community hospitals with limited on-site antimicrobial stewardship programs.

## Methods

We conducted a retrospective review of all adult nonhospice patients with confirmed CDI receiving fidaxomicin for at least 24 hours at 10 UPMC community hospitals, May 1, 2018 through August 31, 2019. The University of Pittsburgh Institutional Review Board approved this project.

Rates of fidaxomicin guideline adherence were evaluated overall and by prescriber specialty. Use was guideline adherent if fidaxomicin was used as monotherapy for recurrent, nonfulminant CDI, or for any episode of nonfulminant CDI with approval from staff involved in the hospital’s approval process (ie, ID, GI, antimicrobial stewardship, or P&T). CDI severity was defined according to IDSA/SHEA criteria, with severe disease requiring a serum white blood cell count >15,000 cells/mL or a serum creatinine >1.5 mg/dL. Fulminant disease required hypotension or shock, ileus, or megacolon.^1^ Progress notes were reviewed for treating physicians’ categorization of severity.

We used descriptive statistics, including means and medians for continuous measures, frequencies for count data, and standard deviations and interquartile ranges for variance. This was a convenience sample using UPMC community hospitals on a shared electronic medical record. All eligible patients during the specified period were included in the analysis.

## Results

We identified 104 unique hospital encounters from 10 community hospitals. Patient and hospital characteristics are displayed in Supplementary Tables 1 and 2 (online). The updates to UPMC guidelines were communicated to pharmacy directors and local stewardship programs at all UPMC hospitals. They were responsible for dissemination of the updates to local providers. Guideline updates were also available on our intranet. Average fidaxomicin use remained low but increased 30% from January 2018–March 2018 (0.65 days of therapy per 1,000 patient days) to May 2018–August 2019 (0.85 days of therapy per 1,000 patient days).

Table 1 describes treatment by CDI episode and provides nonadherence rates by specialty. Most cases receiving fidaxomicin were severe (43%) or fulminant (35%), and ID staff were responsible for 53% of prescribed fidaxomicin. Overall, 52 (50%) of 104 fidaxomicin regimens were nonadherent to UPMC guidelines. Reasons for nonadherence included use for fulminant CDI (n = 36), concurrent use with other CDI treatment (n = 29), and use for initial CDI without local approval (n = 6). ID accounted for 25 of 52 instances of nonadherence.

**Table 1.** Summary of Fidaxomicin Treatment Regimens Stratified by *Clostridioides difficile* Infection (CDI) Episode and Guideline Nonadherence

Variable	First Episode (n = 30), No. (%)	Second Episode (n = 36), No. (%)	Third or Greater Episode (n = 38), No. (%)	Overall (n = 104), No. (%)	Nonadherent Usage (n = 52), No. (%)
**IDSA/SHEA defined CDI severity**					
Nonsevere	7 (23)	10 (28)	6 (16)	23 (22)	
Severe	11 (37)	18 (50)	16 (42)	45 (43)	
Fulminant	12 (40)	8 (22)	16 (42)	36 (35)	
**Fidaxomicin prescribing service**					
ID Medicine	14 (47)	18 (50)	23 (61)	55 (53)	25 (48)
GI	5 (17)	7 (19)	5 (13)	17 (16)	6 (12)
Hospitalist	8 (27)	7 (19)	1 (3)	16 (15)	9 (17)
Critical care	3 (10)	3 (8)	7 (18)	13 (13)	8 (15)
Surgery	0 (0) 0 (0)	1 (3) 0 (0)	1 (3) 1 (3)	2 (2) 1 (1)	2 (4) 2 (4)

**Fig. 1:**
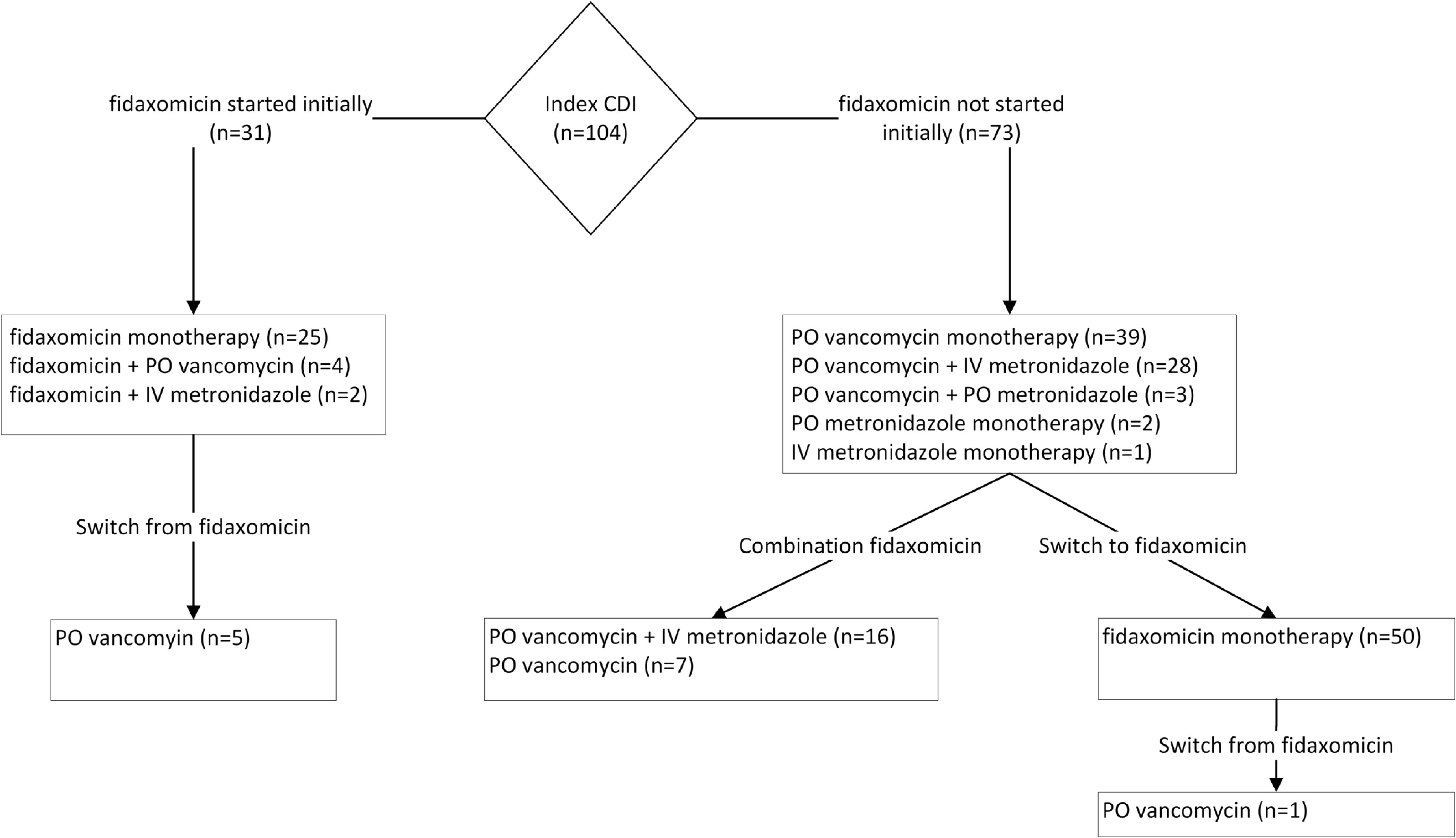
Breakdown of treatment courses involving fidaxomicin.

Overall, 25 patients (24%) received fidaxomicin only. The remaining received a combination (either serial or concurrent) of fidaxomicin, oral vancomycin and/or metronidazole (Fig. 1). The most frequently documented reason for concurrent or serial fidaxomicin use was ongoing diarrhea (n = 21 and n = 45, respectively). ID staff prescribed most of these regimens (n = 12 and n = 33, respectively). Median time from start of initial therapy to fidaxomicin (concurrent or serial) was 3 days; 43% were changed to fidaxomicin within 1 day of the initial regimen.

Of 30 patients in this review treated for initial CDI, 2 had recurrence within 2 months of completing 10 days of fidaxomicin. Both had severe disease treated with oral vancomycin before transitioning to fidaxomicin after ongoing diarrhea. Overall, the median duration of fidaxomicin therapy was 10 (IQR, 5–11) days, and 17 (16%) received fidaxomicin for >10 days. In addition, 100 patients (96.6%) survived to discharge.

## Discussion

We report fidaxomicin prescribing patterns after changes to institutional CDI guidelines. UPMC broadened criteria for fidaxomicin use in 2018 but did not recommend it for initial CDI. This factor, along with lack of awareness of treatment guideline changes, likely contributed to less than one-third of our fidaxomicin use being for initial CDI. However, we also identified unexpected practices, particularly by ID specialists. Overall adherence to our guidelines was only 50%, and ID prescribed about half of these nonadherent regimens. The most frequent nonadherence reasons were for fulminant CDI and use concurrently with other agents. Neither practice was recommended at the time by IDSA/SHEA or UPMC guidelines.

Another unexpected finding was the prescribers’ frequent decision to change treatment soon after starting therapy. The primary documented reason for therapy change was ongoing diarrhea, with 43% of these therapy changes within 1 day of starting the initial regimen. Changes in treatment are often at the discretion of the prescribing physician because treatment guidelines do not provide a standardized period in which symptoms should improve nor do they specify the extent of improvement. Treatment guidelines suggest that 10 days of therapy should be sufficient in most patients with initial CDI but that some patients may have a delayed response.^1^ One trial comparing fidaxomicin to oral vancomycin reported median time to resolution of diarrhea among inpatients to be 2.4 days and 3.2 days, respectively (nonsignificant difference).^5^ Another also demonstrated nonsignificant differences in median time to resolution of diarrhea between fidaxomicin and oral vancomycin (2.3 days vs 2.4 days, respectively).^6^ Switching during early treatment based solely on continuation of diarrhea is not supported by available literature.

We provide valuable information regarding prescribing practices of fidaxomicin in 10 community hospitals belonging to a single health system after a major guideline update. We also reveal the importance of antimicrobial stewardship programs in complementing ID consultants. Strong ID physician leadership in antimicrobial stewardship has been associated with improved patient outcomes and lower antimicrobial use.^7,8^ However, the role of ID physician leadership in antimicrobial stewardship is not synonymous with ID consultation, and this distinction is well described in the literature.^7^ One study demonstrated that 80% of patients in an acute-care hospital accounting for the top 1% of drug expenditures were followed by ID consulting services, and more than half of these had opportunities for cost-effective stewardship interventions.^9^ Antimicrobial stewardship programs should collaborate with ID consultants in coordinating optimal, cost-effective, and guideline-adherent pharmacotherapy.

Our scope was limited to the inpatient setting, but other inpatient and outpatient areas involving ID consultants may be targets for antimicrobial stewardship initiatives. Although release of new or updated clinical guidelines provides opportunities to advance patient care, dissemination of this information is often slow. Antimicrobial stewardship programs play a critical role in dissemination and should ensure that all providers, including ID staff, exercise appropriate stewardship in their prescribing practices.

## Supplementary material

For supplementary material accompanying this paper visit https://doi.org/10.1017/ice.2021.456. click here to view supplementary material
