# Supplementary material for: Evaluation of fidaxomicin use in community hospitals after a clinical guideline change at a large health system and opportunities for stewardship
Source: Infect Control Hosp Epidemiol. 2022 Jan 24;44(2):312–4. doi: 10.1017/ice.2021.456 (PMC9929706; doi:10.1017/ice.2021.456)
Supplement: Supplementary file 1 [file S0899823X21004566sup001.docx]

Supplemental Table 1. Characteristics of Included Patients

|  | N = 104 |
| --- | --- |
| Mean (SD) age in years | 69 (15) |
| Male sex | 64 (62) |
| Index CDI^a^ episodes  First  Second  Third or greater | 30 (29)  36 (35)  38 (37) |
| Recent CDI^a^ (within 30 days of index CDI) | 27 (26) |
| Mean CCI^b^ score (SD) | 5 (3) |
| CCI^b^ category  Low  Medium  High  Very high | 8 (9)  13 (14)  10 (11)  62 (67) |
| Non-CDI^a^ antibiotic exposure  Within 90 days prior to index infection  During current index infection | 32 (31)  20 (19) |
| Other pertinent diagnoses/risk factors for disease  Median (IQR) length of hospitalization in days  Proton Pump Inhibitor Use  Inflammatory bowel disease  Tube Feeds  Active chemotherapy  Solid organ transplant  Hematopoietic Stem Cell Transplant  Neutropenia  Mean serum albumin g/dL | 8 (6)  58 (56)  23 (22)  10 (10)  10 (10)  2 (2)  0 (0)  0 (0)  2.6 (0.8) |

Data presented as n (%) unless otherwise specified

a *Clostridioides difficile* infection

b Charlson comorbidity index

Supplemental Table 2 Hospital Characteristics

| Hospital | Licensed beds | Number of included Cases | ID physician present and involved with ASP | GI physician present | Pharmacist FTE for Stewardship duties | ID trained clinical pharmacist  present | Average hospital onset *Clostridioides difficile* infections per month |
| --- | --- | --- | --- | --- | --- | --- | --- |
| 1 | 394 | 29 | Y | Y | 0.5 | N | 4.5 |
| 2 | 495 | 27 | N | Y | 0.0 | N | 3.5 |
| 3 | 250 | 14 | Y | Y | 1.0 | Y | 3.2 |
| 4 | 156 | 12 | Y | Y | 0.5 | N | 2.9 |
| 5 | 133 | 8 | Y | Y | 0.5 | N | 3.4 |
| 6 | 335 | 5 | Y | Y | 0.25 | N | 1.7 |
| 7 | 213 | 4 | Y | Y | 0.3 | N | 0.6 |
| 8 | 49 | 2 | N | N | 0.0 | N | 0.1 |
| 9 | 228 | 2 | N | Y | 0.0 | N | 1.0 |
| 10 | 171 | 1 | N | Y | 0.5 | N | 0.9 |
